# Associations of BMI with COVID-19 vaccine uptake, vaccine effectiveness, and risk of severe COVID-19 outcomes after vaccination in England: a population-based cohort study

**DOI:** 10.1016/S2213-8587(22)00158-9

**Published:** 2022-08

**Authors:** Carmen Piernas, Martina Patone, Nerys M Astbury, Min Gao, Aziz Sheikh, Kamlesh Khunti, Manu Shankar-Hari, Sharon Dixon, Carol Coupland, Paul Aveyard, Julia Hippisley-Cox, Susan A Jebb

**Affiliations:** a Nuffield Department of Primary Care Health Sciences, Radcliffe Observatory Quarter, University of Oxford, Oxford, UK; b Department of Biochemistry and Molecular Biology II, Faculty of Pharmacy, Center for Biomedical Research, University of Granada, Granada, Spain; c NIHR Oxford Biomedical Research Centre, Oxford University Hospitals, NHS Foundation Trust, Oxford, UK; d Usher Institute, University of Edinburgh, Edinburgh, UK; e Centre for Inflammation Research, University of Edinburgh, Edinburgh, UK; f Diabetes Research Centre, University of Leicester, Leicester, UK; g School of Medicine, University of Nottingham, Nottingham, UK

## Abstract

**Background:**

A high BMI has been associated with a reduced immune response to vaccination against influenza. We aimed to investigate the association between BMI and COVID-19 vaccine uptake, vaccine effectiveness, and risk of severe COVID-19 outcomes after vaccination by using a large, representative population-based cohort from England.

**Methods:**

In this population-based cohort study, we used the QResearch database of general practice records and included patients aged 18 years or older who were registered at a practice that was part of the database in England between Dec 8, 2020 (date of the first vaccination in the UK), to Nov 17, 2021, with available data on BMI. Uptake was calculated as the proportion of people with zero, one, two, or three doses of the vaccine across BMI categories. Effectiveness was assessed through a nested matched case-control design to estimate odds ratios (OR) for severe COVID-19 outcomes (ie, admission to hospital or death) in people who had been vaccinated versus those who had not, considering vaccine dose and time periods since vaccination. Vaccine effectiveness against infection with SARS-CoV-2 was also investigated. Multivariable Cox proportional hazard models estimated the risk of severe COVID-19 outcomes associated with BMI (reference BMI 23 kg/m^2^) after vaccination.

**Findings:**

Among 9 171 524 participants (mean age 52 [SD 19] years; BMI 26·7 [5·6] kg/m^2^), 566 461 tested positive for SARS-CoV-2 during follow-up, of whom 32 808 were admitted to hospital and 14 389 died. Of the total study sample, 19·2% (1 758 689) were unvaccinated, 3·1% (287 246) had one vaccine dose, 52·6% (4 828 327) had two doses, and 25·0% (2 297 262) had three doses. In people aged 40 years and older, uptake of two or three vaccine doses was more than 80% among people with overweight or obesity, which was slightly lower in people with underweight (70–83%). Although significant heterogeneity was found across BMI groups, protection against severe COVID-19 disease (comparing people who were vaccinated *vs* those who were not) was high after 14 days or more from the second dose for hospital admission (underweight: OR 0·51 [95% CI 0·41–0·63]; healthy weight: 0·34 [0·32–0·36]; overweight: 0·32 [0·30–0·34]; and obesity: 0·32 [0·30–0·34]) and death (underweight: 0·60 [0·36–0·98]; healthy weight: 0·39 [0·33–0·47]; overweight: 0·30 [0·25–0·35]; and obesity: 0·26 [0·22–0·30]). In the vaccinated cohort, there were significant linear associations between BMI and COVID-19 hospitalisation and death after the first dose, and J-shaped associations after the second dose.

**Interpretation:**

Using BMI categories, there is evidence of protection against severe COVID-19 in people with overweight or obesity who have been vaccinated, which was of a similar magnitude to that of people of healthy weight. Vaccine effectiveness was slightly lower in people with underweight, in whom vaccine uptake was also the lowest for all ages. In the vaccinated cohort, there were increased risks of severe COVID-19 outcomes for people with underweight or obesity compared with the vaccinated population with a healthy weight. These results suggest the need for targeted efforts to increase uptake in people with low BMI (<18·5 kg/m^2^), in whom uptake is lower and vaccine effectiveness seems to be reduced. Strategies to achieve and maintain a healthy weight should be prioritised at the population level, which could help reduce the burden of COVID-19 disease.

**Funding:**

UK Research and Innovation and National Institute for Health Research Oxford Biomedical Research Centre.

## Introduction

One in five individuals worldwide are at increased risk of severe clinical outcomes after SARS-CoV-2 infection due to underlying health conditions^1^ and there is now consistent evidence showing that obesity is a significant independent risk factor.^2^, ^3^, ^4^, ^5^, ^6^, ^7^ Although the mechanisms for such observations are unclear, there are several plausible explanations for the adverse outcomes from SARS-CoV-2 and other respiratory viruses in people with obesity. These include fat deposited around the airways that could reduce functional lung capacity, obesity-related proinflammatory states that could exacerbate the pathology of COVID-19, higher viral load and prolonged and increased viral shedding that could affect recovery time, as well as hyperinsulinemia and hyperleptinemia that can impair T cell function.^8^, ^9^, ^10^ In addition, fat in the chest wall and abdomen in people with obesity could make ventilation more difficult, airways more prone to collapse, and could require higher pressures to maintain airways, which can lead to increased ventilation-induced damage.^11^

With COVID-19 vaccines showing high levels of effectiveness against both mild and severe COVID-19 in the general population,^12^ it is of crucial importance that all people at higher risk of severe COVID-19, including people with overweight and obesity, are protected. However, there is evidence that vaccine effectiveness for seasonal influenza, among other infectious diseases, is lower in people with obesity than in people of a healthy weight as obesity compromises the response to vaccinations.^13^ For example, studies have reported a reduced serological response to influenza vaccine in the short-term, a poorer sustained seroconversion, impaired T-cell-mediated immune response, and increased risk of influenza among vaccinated adults with obesity.^13^, ^14^, ^15^ Therefore, there is a need to study the effectiveness of COVID-19 vaccines in adults with obesity so that, if differential responses are detected, alternative risk management strategies for infectious respiratory diseases can be employed in this population. Although almost 82·5% of the UK population had received at least two doses of the COVID-19 vaccines by Dec 31, 2021,^16^ there is currently no information available about the uptake of COVID-19 vaccination across BMI groups.

There are sparse data for COVID-19 vaccine effectiveness in people with obesity at the population level. Early efficacy trials showed no evidence of a difference in short-term efficacy against severe COVID-19 outcomes by subgroup (defined as having obesity *vs* not having obesity), but generally lacked the power needed to detect moderate differences.^17^, ^18^, ^19^ A UK cohort study using primary care data also reported a maintained immune response to vaccination and high levels of effectiveness against symptomatic disease (especially after the second dose) in most clinical risk groups, including those with severe obesity (BMI ≥40kg/m^2^).^20^ However, longer-term evidence from the vaccine rollout is needed across a range of COVID-19 outcomes in all BMI groups, which might reveal the need for targeted vaccine booster programmes. These results could support previous associations observed between obesity and COVID-19-related outcomes.^5^

In this study, we used a large, representative population-based cohort of more than 9 million people in England to examine the association between BMI and COVID-19 vaccine uptake, vaccine effectiveness, and risk of severe COVID-19 outcomes after vaccination.

## Methods

### Study design and participants

In this population-based cohort study, we included data from an anonymised research database of patients from over 1700 general practices in England (QResearch version 45), including demographic information, medical diagnoses, and clinical values (eg, BMI and blood pressure). We linked this with data from the NHS Digital database of positive tests for SARS-CoV-2 infection, the UK National Immunisation Database (NIMS) which included data on the uptake of ChAdOx-nCov19 (AstraZeneca), BNT162b2 (Pfizer–BioNTech), and mRNA1273 (Moderna) vaccines from the) and Hospital Episode Statistics (HES) and death certificates from the UK Office for National Statistics (ONS). We included people aged 18 years or older who were registered at a participating general practice during the study period (Dec 8, 2020, to Nov 17, 2021), with at least one BMI measurement in the medical record. Participants were excluded from the study if they had received COVID-19 vaccines before the study start (Dec 8, 2020), if their vaccination date was missing, or if they had a recorded SARS-CoV-2 infection or hospital admission before the study start (appendix 1 p 3).

**Research in context**

**Evidence before this study**

We searched PubMed, medRxiv, and government websites (ie, Public Health England) with the search terms “vaccine effectiveness”, “vaccine uptake”, “obesity”, and “COVID-19” for articles published between Dec 8, 2020 and Dec 31, 2021. We found no information available about the uptake of COVID-19 vaccination among people with overweight and obesity. People with excess weight are at higher risk of severe COVID-19 disease and there are concerns that vaccine effectiveness might also be reduced in this population. Evidence of vaccine effectiveness in relation to BMI is needed to inform decisions about future vaccination policy.

**Added value of this study**

Evidence from this large population-based sample of the English population shows that COVID-19 vaccines were highly protective against severe outcomes when comparing data of people who are vaccinated with that of those who are not vaccinated in all BMI categories. However, vaccines appeared slightly less effective in people who had underweight, who were also less likely to be vaccinated than people in other BMI categories. When investigating COVID-19 outcomes in the vaccinated cohort, people with underweight and those with obesity remained at greater risk of hospitalisation or death from COVID-19 than people with healthy weight, even after a second dose of the vaccine.

**Implications of all the available evidence**

Two doses of COVID-19 vaccines provide a high level of protection against severe COVID-19 outcomes compared with no vaccination across all BMI groups. However, even after vaccination, there were significantly higher risks of severe COVID-19 in people with lower and higher BMIs compared with a healthy BMI. Future research should examine whether these associations persist after booster doses. These results suggest the need for targeted efforts to increase uptake in people with low BMI, in whom uptake is lower and vaccine effectiveness seems to be reduced. Strategies to achieve and maintain a healthy weight should be prioritised at the population level, which might help reduce the burden of COVID-19 disease.

The QResearch database is an anonymised medical research database and was approved by the East Midlands Derby Research Ethics Committee (reference 18/EM/0400). In accordance with this ethical approval, the protocol for this study was reviewed by the QResearch Scientific Advisory Committee before providing approval to access to the data for this project.

### Procedures

BMI (kg/m^2^) was taken from the general practice medical records and we used the last measured BMI before study entry for each individual. We grouped BMI into four categories using the WHO and UK National Institute for Health and Care Excellence (NICE) classification,^21^ with adjustments for Asian ethnicity:^22^ underweight (<18·5 kg/m^2^); healthy weight (18·5–24·9 kg/m^2^ [18·5–22·9 kg/m^2^ for Asian ethnicity]); overweight (25·0–29·9 kg/m^2^ [23–27·5 kg/m^2^ for Asian ethnicity]); and obesity (≥30·0 kg/m^2^ [≥27·5 kg/m^2^ for Asian ethnicity]). We grouped age by 18–39 years, 40–59 years, 60–79 years, and 80 years or older because vaccine rollout in England occurred predominantly by age group. We defined vaccination status for the effectiveness analyses considering vaccine dose and time periods since vaccination as follows: unvaccinated; 0–6 days after first dose; 7–13 days after first dose; 14–20 days after first dose; 21–28 days after first dose; more than 28 days after first dose; 0–6 days after second dose; 7–13 days after second dose; 14 days or more after second dose; 0–6 days after third dose; 7–13 days after third dose; and 14 days or more after third dose.

We analysed the following demographic confounders: age (continuous), sex, self-reported ethnicity (classified as White, Asian, Black, Chinese, and other ethnic groups), socioeconomic status (classified in quintiles of the Townsend score^23^ for individual participants), geographical region, smoking status (divided into never smoked, ex-smoker, light smoker [1–9 cigarettes per day], moderate smoker [10–19 cigarettes per day], and heavy smoker [≥20 cigarettes per day]), and relevant comorbidities (hypertension, cardiovascular disease [including congestive heart failure and stroke], type 1 diabetes, and type 2 diabetes), and care home status (as a binary variable). Missing or not recorded data on ethnicity, socioeconomic status, and smoking status were coded as “unknown” and entered as a separate category.

### Outcomes

For vaccine uptake, the main outcomes of interest were one, two, or three doses of COVID-19 vaccines administered during the study period. For vaccine effectiveness, the main outcomes of interest were hospital admission and death. Hospital admission was defined as having an International Classification of Diseases 10th edition (ICD-10) code in their hospital record for either confirmed (U07.1) or suspected COVID-19 (U07.2) as primary or secondary cause or new hospital admission associated with a confirmed SARS-CoV-2 infection in the preceding 14 days, and death was defined using ICD-10 codes on ONS death certificates for confirmed or suspected death from COVID-19 (primary or secondary cause) within 28 days of a laboratory-confirmed SARS-CoV-2 infection. We also studied an outcome of laboratory-confirmed SARS-CoV-2 infection; however, since variability in testing was expected and a high proportion of asymptomatic infections might not have been detected since vaccination started, the hospital admission and death outcomes were considered more robust outcomes than infection. For risk of COVID-19 after vaccination, we used the same outcomes as the vaccine effectiveness analyses.

### Statistical analysis

We followed a prespecified statistical analysis plan, but we made an amendment to this plan to include the third dose of the vaccine in the analysis since these data became available after the analysis had started (appendix 2). We calculated the proportion of the cohort being vaccinated with one, two, or three doses by BMI and age groups. We used a Cox regression analysis with follow-up time (days from Dec 8, 2020) as the timescale to calculate adjusted hazard ratios (HRs; 95% CI) of receiving first, second, and third dose of vaccine by BMI group, with healthy weight (ie, BMI 18·5–24·9) as the reference category. The model was stratified in 10-year age bands and adjusted for age (continuous), sex, ethnicity, smoking status, socioeconomic status, region, relevant comorbidity, and care home status. Participants entered the analysis on Dec 8, 2020, and were censored on the earliest of date of vaccination, death, or the latest date that data were available. The proportional hazards assumption was met on inspection of log-log plots.

For the vaccine effectiveness analyses, we used a nested matched case-control design to estimate odds ratios [ORs] with 95% CIs for each of the COVID-19 outcomes in people who were vaccinated versus people who were non-vaccinated (appendix 1 p 2). Each participant with a COVID-19 outcome was exactly matched by age, sex, calendar date, general practice, region, and care home status to controls without evidence of a COVID-19 outcome on that date, with a predetermined ratio of 1:10 (cases:controls) drawn from the entire population using incidence density sampling with replacement. Participants entered the analyses on Dec 8, 2020, and were censored on the earliest date of the outcome of interest (ie, COVID-19-related hospital admission, death, or infection), death from other causes, or the latest date for which data were available. Conditional logistic regression models included an interaction between vaccination status and BMI category, and were adjusted for ethnicity, socioeconomic status, smoking status, and comorbidities. Likelihood ratio tests were computed to calculate p values for heterogeneity across BMI groups.

The risk of severe COVID-19 outcomes associated with BMI after vaccination was investigated on the sample of people who had received at least one dose of the vaccine. Consistent with previous research,^24^ we used 14 days or more to assess COVID-19 outcomes associated with BMI after vaccination as there is evidence that 14 days is long enough to generate immunity. A multivariable Cox proportional hazard model with follow-up time (days) as the timescale and restricted cubic splines models with five knots were used to examine non-linear associations (HRs [95% CIs]) between BMI (treated as a continuous variable with reference 23 kg/m^2^, as used in a previous study^5^) and severe COVID-19 outcomes. Separate models were used to examine associations between first and second dose; second and third dose; and after third dose. Models were adjusted for age (continuous), sex, calendar week, ethnicity, smoking, socioeconomic status, region, comorbidities, and care home status. BMI is not necessarily measured and recorded in the medical records. As such, misclassification biases might affect associations, especially for people whose BMI measurements were taken many years before exposure to SARS-CoV-2 and whose BMI has changed since this measurement. Hence, we performed a sensitivity analysis, in which we confined the analyses of vaccine effectiveness and risk of severe COVID-19 outcomes after vaccination to those with BMI recorded within 2 years of cohort entry. A second sensitivity analysis excluded people living in care homes to reduce the possibility of reverse causality.

We did all analyses in Stata (version 17).

### Role of the funding source

The funder of the study had no role in study design, data collection, data analysis, data interpretation, or writing of the report.

## Results

From the original sample of 12 155 659 patients (registered in 1738 different general practices participating with QResearch), 9 694 079 patients had BMI data. A total of 9 171 524 adults aged 18 years or older (with a mean of 52 [SD 19] years) with a BMI measurement (mean BMI 26·7 [5·6] kg/m^2^) were included in the main analyses after we excluded those with COVID-19 vaccination dates before the study start date (n=673), missing vaccination dates (n=1491), or SARS-CoV-2 infection before the study start (n=520 391; see the table and appendix 1 [p 3]). There was a mean of 5·6 (SD 5·0) years and a median of 3·9 (IQR 1·7–7·9) years since the BMI measurements were taken before the study started. From Dec 8, 2020, to Nov 17, 2021, a total of 566 461 positive tests for SARS-CoV-2 infection, 32 808 hospital admissions from COVID-19, and 14 389 deaths from COVID-19 were recorded in the cohort. The demographic characteristics of the sample, overall and by BMI and vaccination status, are shown in table and appendix 1 (pp 4–5).

**Table:** Baseline characteristics of the study population by BMI

		**Total Population**	**Underweight (<18·5 kg/m^2^)**	**healthy weight (18·5 to 24·9 kg/m^2^)**	**Overweight (25·0 to 29·9 kg/m^2^)**	**Obesity (≥30·0 kg/m^2^)**
Total sample, n		9 171 524	320 737	3 509 213	3 062 925	2 278 649
Deaths from COVID-19		14 389 (0·2%)	461 (0·1%)	4001 (0·1%)	4688 (0·2%)	5239 (0·2%)
Hospital admission from COVID-19		32 808 (0·4%)	796 (0·2%)	8315 (0·2%)	10 653 (0·3%)	13 044 (0·6%)
Positive tests for COVID-19		566 461 (6·2%)	22 403 (7·0%)	215 852 (6·2%)	180 380 (5·9%)	147 826 (6·5%)
Vaccine doses						
	Unvaccinated	1 758 689 (19·2%)	104 488 (32·6%)	817 741 (23·3%)	513 570 (16·8%)	322 890 (14·2%)
	One	287 246 (3·1%)	20 303 (6·3%)	121 348 (3·5%)	82 473 (2·7%)	63 122 (2·8%)
	Two	4 828 327 (52·6%)	163 844 (51·1%)	1 851 750 (52·8%)	1 589 893 (51·9%)	1 222 840 (53·7%)
	Three	2 297 262 (25·0%)	32 102 (10·0%)	718 374 (20·5%)	876 989 (28·6%)	669 797 (29·4%)
Mean BMI (SD), kg/m^2^		26·7 (5·6)	17·2 (0·9)	22·2 (1·7)	27·1 (1·5)	34·3 (4·1)
Type 2 diabetes		694 218 (7·6%)	3854 (1·2%)	98 830 (2·8%)	231 604 (7·6%)	359 930 (15·8%)
Type 1 diabetes		59 826 (0·7%)	1321 (0·4%)	20 434 (0·6%)	20 862 (0·7%)	17 209 (0·8%)
Cardiovascular disease		605 585 (6·6%)	9145 (2·9%)	152 770 (4·4%)	235 142 (7·7%)	208 528 (9·2%)
Hypertension		1 792 284 (19·5%)	16 696 (5·2%)	371 401 (10·6%)	665 563 (21·7%)	738 624 (32·4%)
Mean age (SD), years		52 (19)	37 (19)	48 (19)	55 (18)	55 (17)
Sex						
	Men	4 312 323 (47·0%)	140 307 (43·7%)	1 494 345 (42·6%)	1 660 888 (54·2%)	1 016 783 (44·6%)
	Women	4 859 201 (53·0%)	180 430 (56·3%)	2 014 868 (57·4%)	1 402 037 (45·8%)	1 261 866 (55·4%)
Ethnicity						
	White	6 070 653 (66·2%)	175 820 (54·8%)	2 354 826 (67·1%)	2 038 132 (66·5%)	1 501 875 (65·9%)
	Asian	676 214 (7·4%)	33 182 (10·3%)	165 517 (4·7%)	256 658 (8·4%)	220 857 (9·7%)
	Black	320 681 (3·5%)	9635 (3·0%)	99 777 (2·8%)	1 11 557 (3·6%)	99 712 (4·4%)
	Chinese	82 387 (0·9%)	8220 (2·6%)	55 113 (1·6%)	15 363 (0·5%)	3691 (0·2%)
	Other or not recorded	2 021 589 (22·0%)	93 880 (29·3%)	833 980 (23·8%)	641 215 (20·9%)	452 514 (19·9%)
Quintile of Townsend						
	1	2 267 236 (24·7%)	61 743 (19·3%)	868 987 (24·8%)	823 146 (26·9%)	513 360 (22·5%)
	2	2 008 005 (21·9%)	59 574 (18·6%)	745 584 (21·2%)	7 00 687 (22·9%)	502 160 (22·0%)
	3	1 770 337 (19·3%)	61 391 (19·1%)	651 264 (18·6%)	583 646 (19·1%)	474 036 (20·8%)
	4	1 586 007 (17·3%)	65 087 (20·3%)	603 718 (17·2%)	494 111 (16·1%)	423 091 (18·6%)
	5	1 498 195 (16·3%)	71 377 (22·3%)	621 864 (17·7%)	448 133 (14·6%)	356 821 (15·7%)
	Missing data or not recorded	41 744 (0·5%)	1 565 (0·5%)	17 796 (0·5%)	13 202 (0·4%)	9181 (0·4%)
Smoking status						
	Non-smoker	5 345 262 (58·3%)	185 677 (57·9%)	2 108 349 (60·1%)	1 763 721 (57·6%)	1 287 515 (56·5%)
	Ex-smoker	2 102 775 (22·9%)	32 969 (10·3%)	668 375 (19·0%)	779 633 (25·5%)	621 798 (27·3%)
	Light smoker	1 204 760 (13·1%)	52 778 (16·5%)	520 548 (14·8%)	372 440 (12·2%)	258 994 (11·4%)
	Moderate smoker	252 775 (2·8%)	10 695 (3·3%)	107 362 (3·1%)	78 195 (2·6%)	56 523 (2·5%)
	Heavy smoker	119 573 (1·3%)	4157 (1·3%)	46 131 (1·3%)	38 443 (1·3%)	30 842 (1·4%)
	Missing data or not recorded	146 379 (1·6%)	34 461 (10·7%)	58 448 (1·7%)	30 493 (1·0%)	22 977 (1·0%)

Data are in n (%) unless specified otherwise.

**Figure 1:**
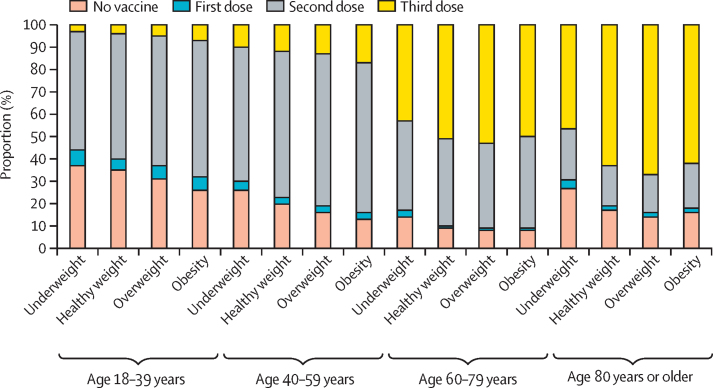
Proportion of people who received no, one, two, or three vaccination doses by age and BMI group Data from Nov 17, 2021.

By Nov 17, 2021, across the entire cohort, 19·2% (1 758 689) of participants were unvaccinated, 3·1% (287 246) had one vaccine dose, 52·6% (4 828 327) had two doses, and 25·0% (2 297 262) had three doses. Uptake of two or three vaccine doses was more than 80% (81–91%) among people with overweight or obesity in the 40–59 years, 60–79 years, and 80 years and older age groups (figure 1, appendix 1 [p 6]), but was slightly lower in people classified as underweight (from 70–83%) in the same age groups. Compared with the healthy weight category, the adjusted HR for receiving the first dose of the vaccine was significantly lower in people with underweight (HR 0·909 [95% CI 0·905–0·913]) but significantly higher in those with overweight (1·120 [1·118–1·122]) and obesity (1·202 [1·199–1·204]; appendix 1 [p 7]). The adjusted HRs for the second dose were similar in participants in all other BMI categories compared with those in the healthy weight category (underweight: 1·044 [1·040–1·049]; overweight: 1·001 [0·999–1·003]; and obesity: 1·003 [1·001–1·005]); whereas the HRs for the third dose followed the same pattern observed in uptake of the first dose (underweight: 0·877 [0·867–0·887]; overweight: 1·043 [1·040–1·047]; and obesity: 1·044 [1·040–1·047]).

**Figure 2:**
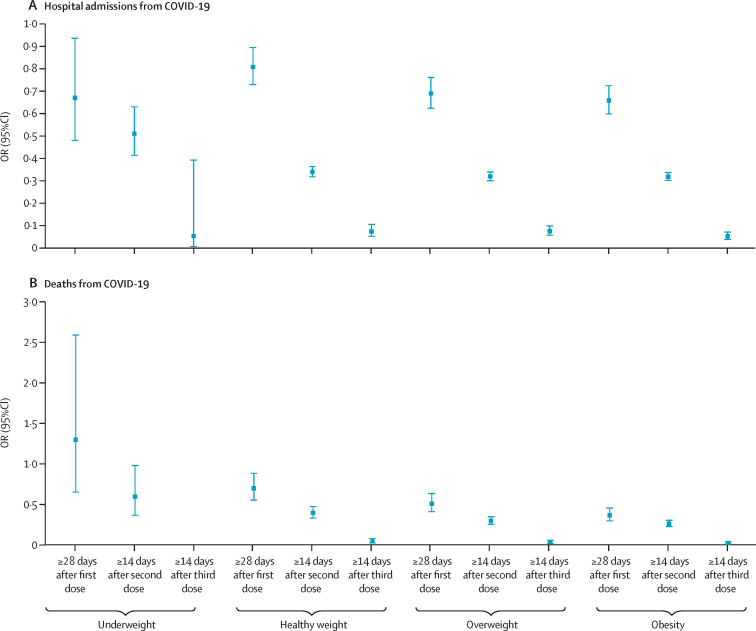
Vaccine effectiveness against hospital admission and death from COVID-19 in vaccinated vs non-vaccinated individuals grouped by BMI status Unvaccinated population was used as baseline for OR (95% CI). Cases and controls were matched by age, sex, calendar date, practice, region, and care home status. Models were additionally adjusted for ethnicity, socioeconomic status, smoking status, hypertension, type 1 diabetes, type 2 diabetes, and cardiovascular disease. There was no estimate for the OR for deaths from COVID-19 for the underweight category (≥14 days after third dose). OR=odds ratio.

The likelihood of severe COVID-19 outcomes (hospital admission or death) by BMI category and interval after vaccination compared with people who were unvaccinated can be found in figure 2 and appendix 1 (pp 8–10). The ORs for hospital admission were reduced in people after the first, second, and third vaccine doses compared with those who were unvaccinated across all BMI categories, although there was significant heterogeneity (P=0·0010). 14 days after the second dose, there was a significantly lower likelihood of hospital admission compared with those who were not vaccinated (underweight: OR 0·51 [CI 95% 0·41–0·63]; healthy weight: 0·34 [0·32–0·36]; overweight: 0·32 [0·30–0·34]; and obesity: 0·32 [0·30–0·34]); as well as after 14 days from the third dose (underweight: 0·05 [0·01–0·39]; healthy weight: 0·07 [0·05–0·11]; overweight: 0·08 [0·06–0·10]; and obesity: 0·05 [0·04–0·07]).

The ORs for death followed a similar pattern to those for hospitalisation, with a significantly lower likelihood of death in participants who were vaccinated versus not vaccinated and significant heterogeneity by BMI category (P<0·0001). ORs after 14 days from the second dose were 0·60 (95% CI 0·36–0·98) in participants with underweight; 0·39 (0·33–0·47) in those with healthy weight, 0·30 (0·25–0·35) in those with overweight, and 0·26 (0·22–0·30) in those with obesity. The ORs for death after the third dose were also significantly reduced compared with participants who were unvaccinated, although there was more uncertainty due to a much lower number of cases for this outcome (underweight: no cases; healthy weight: 0·04 [0·02–0·08]; overweight: 0·03 [0·02–0·06]; and obesity: 0·02 [0·01–0·04]).

There was also a significantly higher likelihood of confirmed SARS-COV-2 infection after the first and second doses in participants who had been vaccinated versus those who had not, but lower after the third dose with significant heterogeneity by BMI category (P<0·0001; appendix 1 [p 10]).

**Figure 3:**
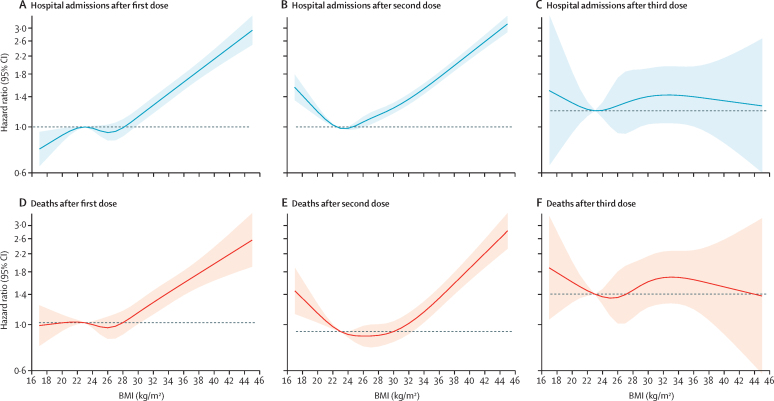
Risk of severe outcomes from COVID-19 after vaccination Estimates of risk after 14 days from each vaccine dose. Adjusted for age, calendar week, sex, ethnicity, socioeconomic status, region, smoking status, hypertension, type 1 diabetes, type 2 diabetes, cardiovascular disease, and care home status. Hospital admissions from COVID-19 after first dose (A), second dose (B), and third dose (C), and deaths from COVID-19 after first dose (D), second dose (E), and third dose (F).

In people who had at least one vaccine (n=7 412 835), the risk of hospital admission or death increased linearly with BMI after about 30 kg/m^2^ compared with a BMI of 23 kg/m^2^, but there was no evidence that risk significantly increased in those with a BMI of less than 30 kg/m^2^ (figure 3). After the second dose, there were clear J-shaped associations between BMI and COVID-19-related hospitalisation and death, with significantly higher HRs at very low (eg, 18 kg/m^2^) and very high BMIs (eg, 40 kg/m^2^) compared with BMIs of 23 kg/m^2^. After the third dose, the number of cases of hospitalisation and death was much smaller and there was little evidence of any association with BMI with wide 95% CIs.

For COVID-19 test positivity (appendix 1 p 11), there was a linear association with BMI after the first dose, an exponential association after the second dose, and inverse U-shaped association after the third dose with significantly lower HRs at very low and very high BMI levels.

Sensitivity analyses of estimates of vaccine effectiveness and risk of severe COVID-19 outcomes after vaccination generally supported the main study conclusions (appendix 1 pp 12–15). The first set of sensitivity analyses were calculated including only those with BMI recorded within 2 years before cohort entry (n=2 737 610; 30% of total sample; appendix 1 pp 12–14)). Vaccine effectiveness in this sensitivity analysis generally showed smaller ORs compared with the main analysis; however, the shape of the associations between BMI and risk of severe COVID-19 outcomes in the vaccinated cohort were generally consistent. However, the sample of people with a BMI measurement within the previous 2 years before study start date presented a different distribution of participants within each BMI group (underweight: 2·3%; healthy weight: 31·3%; overweight: 33·6%; and obesity: 32·8%) compared with the main sample (underweight: 3·5%; healthy weight: 38·3%; overweight: 33·4%; and obesity: 24·8%). A second sensitivity analysis excluded people living in care homes (n=9 125 761, 99% of total sample) and was consistent with the main results (appendix 1 pp 13–15).

## Discussion

This large population-based cohort analysis provides evidence that the effectiveness of COVID-19 vaccines against severe outcomes is high. We assessed effectiveness by comparing people who had been vaccinated with those who had not been vaccinated across all BMI categories. Vaccine protection was slightly lower in people with underweight, who were also less likely to be vaccinated. When investigating severe COVID-19 outcomes in the vaccinated cohort, people with underweight and those with obesity remained at greater risk of hospitalisation or death from COVID-19 than people who are a healthy weight, even after a second dose of the vaccine.

A major strength of this study is the inclusion of a large representative sample of the English population, with linkages to vaccination and COVID-19 records (up to Nov 17, 2021). However, some of the estimates (especially after the third dose) were limited by the small number of events. The study also had limited statistical power to investigate the differences in effectiveness across vaccine brands or virus strains. We used a matched case-control design to investigate effectiveness. A negative-test case-control design would be a more robust method, especially for infection outcomes, to control for factors that are usually not measured such as health-seeking behaviours in people who were vaccinated versus those who were unvaccinated, access to testing, and case ascertainment.^25^, ^26^ However, data on negative test results were not available in the QResearch database we used. Another limitation was that outcome misclassification, especially for hospital admissions that happened at the peak of each wave, was possible given that people might have presented with severe clinical illness and it was not possible to be certain whether this admission was due to their medical condition or whether their medical condition was worsened by the SARS-CoV-2 infection. With regards to the exposure, BMI was only available for 79·7% of the original Qresearch population, which could have introduced some degree of bias considering that our previous study reported that people without a recorded BMI were younger, less likely to have comorbidities, and showed a stronger association with death from COVID-19 than those with a recorded BMI.^5^ Another a limitation, as in any study using BMI measurements, is that measurements of height and weight can be imprecise, with some measurements made by a health-care professional and others self-reported (particularly during the pandemic). There will also be daily fluctuations and errors in BMI measurement caused by clothing, which we were unable to account for in this analysis. Some measurements of BMI were recorded many years before study entry, but our sensitivity analyses of vaccine effectiveness and risk of outcomes after vaccination were robust when restricted to people with BMI measurements within the previous 2 years from the study start date. It was not possible to account for changes in weight that might have occurred during the pandemic as the reduction in face-to-face contacts means that weight was unlikely to be independently measured during this period. However, evidence suggests that these fluctuations were small and consistent with prepandemic trends in adult bodyweight.^27^, ^28^ Finally, as with any observational analyses, there was a possibility of residual confounding due to unmeasured covariates (eg, treatment with corticosteroids, presence of other comorbidities, population density, occupation, or related health behaviours such as physical activity).

At a population level, vaccine effectiveness depends on uptake. For other respiratory diseases, such as influenza, a substantially lower uptake of vaccines among people with obesity has been reported in the UK compared to those without obesity. In the UK, 39% of people younger than 65 years with a BMI of 40 kg/m^2^ or more received the influenza vaccine in 2017–18 compared with an average of 49% in this age group.^29^ Conversely, we found higher uptake of two or three vaccine doses among people with overweight and obesity than among those with healthy weight, but uptake was significantly lower among those with underweight. The higher vaccine coverage found among people with obesity might be because the UK used risk-based scheduling that prioritised people with a BMI of 40 kg/m^2^ or more over people in comparator age groups for vaccinations.^30^ In addition, scheduling prioritised people with weight-related conditions (eg, type 2 diabetes) for early vaccination.^30^ However, adjusting for comorbidities did not explain the higher uptake of vaccine doses. The lower vaccine uptake among people with underweight in all age groups was not fully explained by the included demographic or clinical factors, but we did not adjust for other conditions (eg, cancer, autoimmune, or mental health diseases), which could affect vaccine uptake.

In the UK, the most widely used COVID-19 vaccines, ChAdOx-nCov19, BNT162b2, and mRNA1273, have shown high levels of effectiveness against mild and severe disease in the general population after the second dose,^12^ and after a booster dose with BNT162b2.^31^ A UK cohort study (of approximately 7 million participants) using primary care data reported a maintained immune response to vaccination and high levels of effectiveness against symptomatic disease after the second dose in most clinical risk groups.^20^ Vaccine effectiveness for people with a BMI of 40 kg/m^2^ or more was reported to be 86% after 14 days from the second dose of any vaccine, which was consistent with the early phase 3 trial results of the mRNA vaccines among people with obesity.^17^, ^19^ In this Article, we have shown 40–74% lower odds of hospital admission or death after the second dose in all BMI categories (with slightly lower effectiveness estimates in people with underweight) and more than 90% lower odds after the third dose; however, these estimates need to be interpreted with caution due to the smaller number of cases and shorter follow-up periods after the third dose. The reduced effectiveness observed in the underweight group might be explained by the presence of frailty or underlying conditions that were not adjusted in the models, such as cancer or other diseases associated with immunosuppression, as those conditions have been associated with reduced seroconversion and antibody responses, especially after only one dose.^20^, ^32^

Surprisingly, we observed a higher risk of test positivity after vaccination with one or two doses across all BMI groups, which is contrary to evidence reported by the UK ONS.^33^ This finding might be spurious as we could not use a negative test case-control design to assess vaccine effectiveness and could not control for factors such as health-seeking behaviours, access to testing, and case ascertainment in people who have been vaccinated versus people who have not been vaccinated.^25^, ^26^ There might also have been a high proportion of asymptomatic cases that were not being tested (hence not recorded in our data) after vaccination.

When our analyses were restricted to the vaccinated cohort, we observed an increased risk of severe COVID-19 outcomes in the people with higher and lower BMIs compared with those with a BMI of 23 kg/m^2^, particularly after the second dose of the vaccine. The shape of this association is consistent with our previous study in this cohort before the vaccination programme commenced.^5^ Among people with obesity, consistent results have also been reported from a small prospective observational study (n=1022) investigating the risk of influenza among vaccinated individuals, in which, despite robust serological responses, adults with obesity who had been vaccinated were twice as likely to develop influenza than their counterparts with a healthy weight.^13^ It is plausible that, even with a robust serological response, there is an impaired T-cell response^14^, ^15^, ^34^ that could explain the persistent higher risks of severe COVID-19 outcomes associated with obesity despite vaccination. The lack of association observed after the third dose might be an early signal that a booster is needed to confer full protection in people with obesity, but these associations are based on a very small number of cases and need to be re-evaluated in the future. At the lower end of the BMI spectrum, the association with hospital admission and death might be confounded by frailty, which is associated with people with a low BMI^35^, ^36^ (although residual confounding due to cancer or other diseases and treatments cannot be ruled out), which might also explain the apparently reduced effectiveness of the vaccine observed in this BMI group.

In summary, this large community-based cohort of 9 million people in England provides evidence that people with overweight and obesity who are vaccinated are more protected against severe COVID-19. Effectiveness was found to be lower in people classified as underweight, among whom vaccine uptake was also significantly lower. Despite the observed effectiveness of vaccination in people across all BMIs, there were significantly higher risks of severe COVID-19 outcomes in vaccinated people with lower and higher BMIs than in people with a BMI of 23 kg/m^2^, even after the second dose of the vaccine. However, these associations might be reduced after the third dose, which needs to be confirmed in future studies using longer-term follow-up data. These associations, together with the patterns seen after vaccination against influenza, underline the importance of research to better understand the impact of bodyweight on immune function.

## Data sharing

Deidentified individual-participant data reported in this Article will be made available on request for proposals that set out to achieve aims specified in a methodologically and scientifically sound protocol that are approved by the QResearch Scientific Advisory Committee (“learned intermediary”), where costs of providing access to the data are covered, where requests are compliant with the legal permissions of QResearch data providers, and QResearch data security requirements are met. Information regarding submission of applications to access data can be found at www.qresearch.org. Access to the code is available from the authors on request for non-commercial, academic, and research use only.

## Declaration of interests

QResearch is a registered trademark of Egton Medical Information Systems and the University of Nottingham. PA spoke at a symposium at the Royal College of General Practitioners annual conference on interventions for weight loss that was funded by Novo Nordisk but received no personal payments. JH-C received personal fees and other support from ClinRisk (until 2019) outside the submitted work and is an unpaid director of QResearch. MG, NMA, CP, MP, SD, MS-H, CC, and SAJ declare no competing interests. AS is a member of the Scottish Government COVID-19 Chief Medical Officers Advisory Group and its Standing Committee on Pandemics; a member of NERVTAG's Risk Stratification Subgroup; and a member of AstraZeneca's Thrombotic Thrombocytopenic Taskforce. KK is a member of Chair of the Ethnicity Subgroup of the UK Scientific Advisory Group for Emergencies (SAGE) and a member of SAGE.
