# Supplementary material for: Associations of BMI with COVID-19 vaccine uptake, vaccine effectiveness, and risk of severe COVID-19 outcomes after vaccination in England: a population-based cohort study
Source: Lancet Diabetes Endocrinol. 2022 Aug;10(8):571–80. doi: 10.1016/S2213-8587(22)00158-9 (PMC9246477; doi:10.1016/S2213-8587(22)00158-9)
Supplement: Supplementary appendix 1 [file mmc1.pdf]

THE LANCET
Diabetes & Endocrinology

Supplementary appendix 1

This appendix formed part of the original submission and has been peer reviewed. We post it as supplied by the authors.

Supplement to: Piernas C, Patone M, Astbury NM, et al. Associations of BMI with COVID-19 vaccine uptake, vaccine effectiveness, and risk of severe COVID-19 outcomes after vaccination in England: a population-based cohort study. *Lancet Diabetes Endocrinol* 2022; published online June 30. https://doi.org/10.1016/S2213-8587(22)00158-9.

Associations of body mass index with COVID-19 vaccine uptake, vaccine effectiveness and risk of severe COVID-19 outcomes after vaccination: population-based cohort of 9 million people in England

Contents

Supplementary Appendix 1 – Methods 2

Supplementary Appendix 1 – Figures and Tables 3

- Supplementary Figure 1. Study flow diagram. 3
- Supplementary Table 1 – Demographic characteristics by BMI 4
- Supplementary Table 2 – Demographic characteristics by vaccination status 5
- Supplementary Table 3. Percentage of population with one, two or three vaccine doses by BMI and age group 6
- Supplementary Table 4. Cox proportional hazards of vaccination across BMI groups 7
- Supplementary Table 5. Vaccine effectiveness against COVID-19 Hospital admission using a case-control study design 8
- Supplementary Table 6. Vaccine effectiveness against COVID-19 death using a case-control study design 9
- Supplementary Table 7. Vaccine effectiveness against COVID-19 positive test using a case-control study design 10
- Supplementary Figure 2. Risk of COVID-19 positive test after vaccination 11
- Supplementary Figure 3. Sensitivity analysis of vaccine effectiveness against COVID-19 hospital admission and death among people with recent BMI measurements 12
- Supplementary Figure 4. Sensitivity analysis of vaccine effectiveness against COVID-19 hospital admission and death excluding people living in care homes 13
- Supplementary Figure 5. Sensitivity analysis of risk of severe COVID-19 outcomes after vaccination among people with recent BMI measurements 14
- Supplementary Figure 6. Sensitivity analysis of risk of severe COVID-19 outcomes after vaccination excluding people living in care homes 15

Supplementary Appendix 1 – Methods

Supplementary Methods 1 – Case control study design, matching and sample characteristics

For the vaccine effectiveness analyses, a nested matched case-control design was used to estimate odds ratios with 95% confidence intervals (OR and 95% CI) for each of the COVID-19 outcomes in vaccinated versus non-vaccinated people. Each participant with a COVID-19 outcome was exactly matched by age, sex, calendar date, practice, region and care home status to controls without evidence of a COVID-19 outcome on that date, with a predetermined ratio of 1:10 cases to controls drawn from the entire population using incidence density sampling with replacement (a selected control was eligible to be selected again as a control for another case providing this person is free of disease). Participants entered the analyses on December 8, 2020 and were censored on the earliest of date of outcome of interest, death from other causes, or the latest date for which data were available. Conditional logistic regression models included an interaction between vaccination status and BMI categories, and were adjusted for ethnicity, Townsend score, smoking status and co-morbidities. Likelihood ratio tests were computed to determine P values for heterogeneity across BMI groups.

Matching of cases and controls

The matching used predetermined ratio of 1:10 cases to controls. However, the final number of matched controls to each case in each set was as follows:

1. COVID-19 Hospital admission: A total of 276,050 controls (93%) were included in sets of 11 participants; 14,701 controls (5%) were included in sets of 7-10 participants; 7,048 controls (2%) were included in sets of 2-6 participants.

1. COVID-19 Death: A total of 99,200 controls (86%) were included in sets of 11 participants; 9,535 controls (8%) were included in sets of 7-10 participants; 6,040 controls (5%) were included in sets of 2-6 participants.

Characteristics of cases and controls

	COVID-19 Hospital admission		COVID-19 Death	
Characteristics	Cases N=32,808	Controls N=297,799	Cases N=14,389	Controls N=114,775
Age, mean (SD)	66.2 (20.2)	64.3 (19.7)	79.3 (12.7)	77.0 (12.5)
Sex (% men)	50.3	49.9	54.8	55.6
BMI, mean (SD)	28.9 (6.4)	27.2 (5.4)	28.3 (6.3)	27.4 (5.1)
Ethnicity (% Whites)	65.7	67.5	67.5	69.8
Townsend quintile (%)				
1	19.4	24.2	22.5	28.0
3	20.7	19.5	20.7	19.4
5	18.8	16.2	15.1	12.6
Smoking (%)				
Non smoker	40.7	56.1	11.9	55.2
Ex-smoker	24.1	28.3	10.2	33.8
Light smoker	7.7	11.0	2.0	7.6
Moderate smoker	1.5	2.4	0.4	1.6
Heavy smoker	0.8	1.3	0.3	1.0
Not recorded	25.2	0.9	75.1	0.9
Type 2 Diabetes (%)	24.6	13.7	31.9	18.9
Type 1 Diabetes (%)	1.7	0.6	1.6	0.6
Cardiovascular disease (%)	24.7	15.1	37.8	23.6
Hypertension (%)	45.0	36.1	61.0	51.8

Supplementary Appendix 1 – Figures and Tables

Supplementary Figure 1. Study flow diagram.

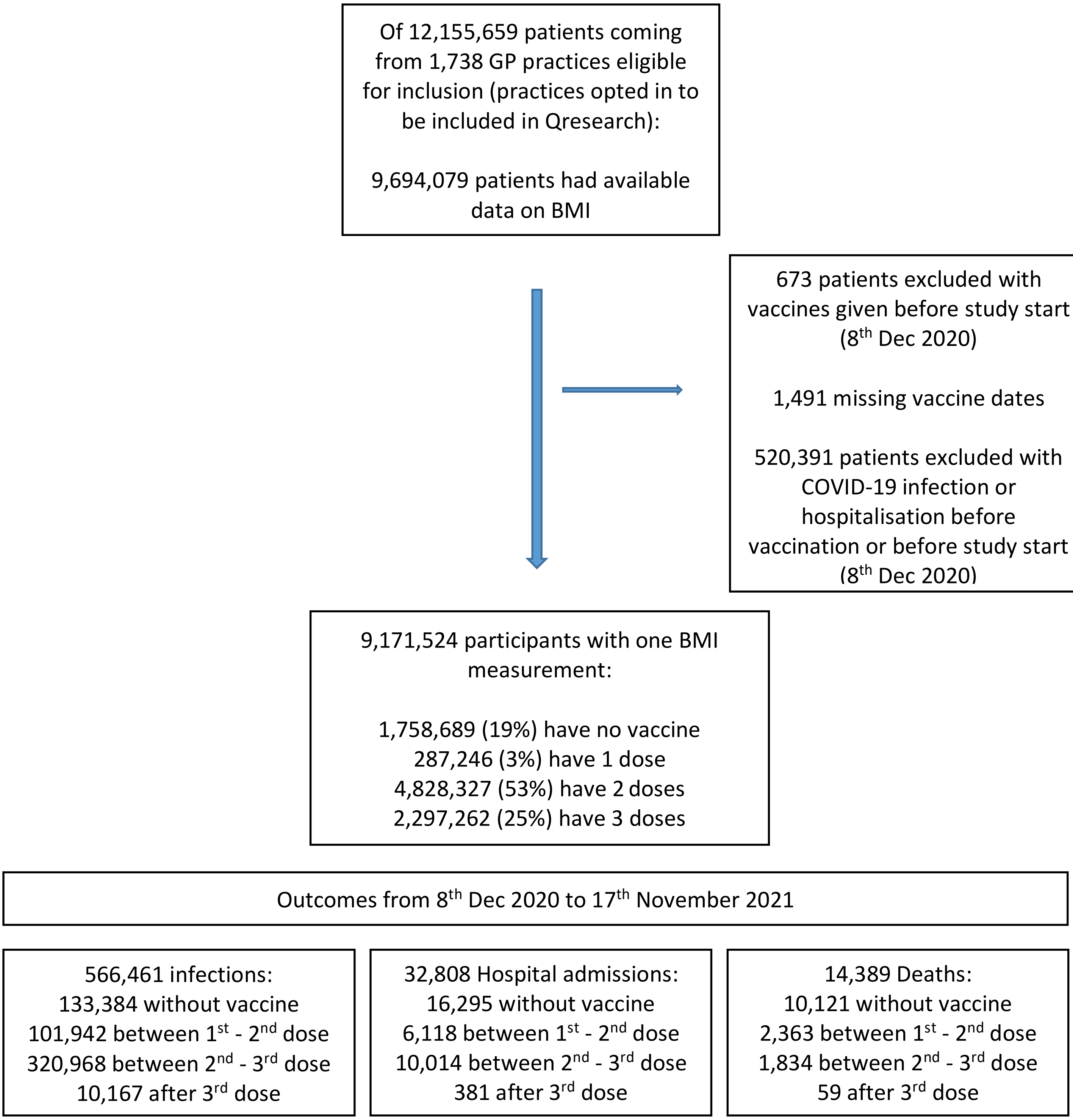

1. Hospital admission: defined as having ICD-10 code in hospital record for either confirmed (U07.1) or suspected COVID-19 (U07.2) as primary or secondary cause, or new hospital admission associated with confirmed COVID-19 within 14 days of the test;
2. Death: defined using ICD-10 codes on ONS death certificates for confirmed or suspected death from COVID-19 (primary or secondary cause) within 28 days of a laboratory confirmed COVID-19 infection.

Supplementary Table 1 – Demographic characteristics by BMI

	Total Population	Underweight (<18.5)	healthy weight (18.5 to <25)	Overweight (25 to <30)	Obesity (30+)
Total sample (n)	9171524	320737	3509213	3062925	2278649
COVID-19 Deaths	14389 (0.2%)	461 (0.1%)	4001 (0.1%)	4688 (0.2%)	5239 (0.2%)
COVID-19 Hospital admission	32808 (0.4%)	796 (0.2%)	8315 (0.2%)	10653 (0.3%)	13044 (0.6%)
COVID-19 Positive test	566461 (6.2%)	22403 (7.0%)	215852 (6.2%)	180380 (5.9%)	147826 (6.5%)
Vaccine doses					
0	1758689 (19.2%)	104488 (32.6%)	817741 (23.3%)	513570 (16.8%)	322890 (14.2%)
1	287246 (3.1%)	20303 (6.3%)	121348 (3.5%)	82473 (2.7%)	63122 (2.8%)
2	4828327 (52.6%)	163844 (51.1%)	1851750 (52.8%)	1589893 (51.9%)	1222840 (53.7%)
3	2297262 (25.0%)	32102 (10.0%)	718374 (20.5%)	876989 (28.6%)	669797 (29.4%)
BMI, mean (SD)	26.7 (5.6)	17.2 (0.9)	22.2 (1.7)	27.1 (1.5)	34.3 (4.1)
Type 2 Diabetes	694218 (7.6%)	3854 (1.2%)	98830 (2.8%)	231604 (7.6%)	359930 (15.8%)
Type 1 Diabetes	59826 (0.7%)	1321 (0.4%)	20434 (0.6%)	20862 (0.7%)	17209 (0.8%)
Cardiovascular disease	605585 (6.6%)	9145 (2.9%)	152770 (4.4%)	235142 (7.7%)	208528 (9.2%)
Hypertension	1792284 (19.5%)	16696 (5.2%)	371401 (10.6%)	665563 (21.7%)	738624 (32.4%)
Age, mean (SD)	52 (19)	37 (19)	48 (19)	55 (18)	55 (17)
Sex (Men)	4312323 (47.0%)	140307 (43.7%)	1494345 (42.6%)	1660888 (54.2%)	1016783 (44.6%)
Ethnicity					
White	6070653 (66.2%)	175820 (54.8%)	2354826 (67.1%)	2038132 (66.5%)	1501875 (65.9%)
Asian	676214 (7.4%)	33182 (10.3%)	165517 (4.7%)	256658 (8.4%)	220857 (9.7%)
Black	320681 (3.5%)	9635 (3.0%)	99777 (2.8%)	111557 (3.6%)	99712 (4.4%)
Chinese	82387 (0.9%)	8220 (2.6%)	55113 (1.6%)	15363 (0.5%)	3691 (0.2%)
Others/not recorded	2021589 (22.0%)	93880 (29.3%)	833980 (23.8%)	641215 (20.9%)	452514 (19.9%)
Quintile of Townsend					
1	2267236 (24.7%)	61743 (19.3%)	868987 (24.8%)	823146 (26.9%)	513360 (22.5%)
2	2008005 (21.9%)	59574 (18.6%)	745584 (21.2%)	700687 (22.9%)	502160 (22.0%)
3	1770337 (19.3%)	61391 (19.1%)	651264 (18.6%)	583646 (19.1%)	474036 (20.8%)
4	1586007 (17.3%)	65087 (20.3%)	603718 (17.2%)	494111 (16.1%)	423091 (18.6%)
5	1498195 (16.3%)	71377 (22.3%)	621864 (17.7%)	448133 (14.6%)	356821 (15.7%)
Missing/not recorded	41744 (0.5%)	1565 (0.5%)	17796 (0.5%)	13202 (0.4%)	9181 (0.4%)
Region					
East Midlands	399276 (4.4%)	17342 (5.4%)	159144 (4.5%)	130138 (4.2%)	92652 (4.1%)
East of England	515578 (5.6%)	18234 (5.7%)	196429 (5.6%)	177451 (5.8%)	123464 (5.4%)
London	2142178 (23.4%)	82666 (25.8%)	893290 (25.5%)	691872 (22.6%)	474350 (20.8%)
North East	294670 (3.2%)	10166 (3.2%)	103092 (2.9%)	97771 (3.2%)	83641 (3.7%)
North West	1509804 (16.5%)	49689 (15.5%)	529315 (15.1%)	512505 (16.7%)	418295 (18.4%)
South Central	1093980 (11.9%)	34728 (10.8%)	420876 (12.0%)	373959 (12.2%)	264417 (11.6%)
South East	897277 (9.8%)	28382 (8.8%)	350972 (10.0%)	302608 (9.9%)	215315 (9.4%)
South West	903423 (9.9%)	29825 (9.3%)	357861 (10.2%)	297544 (9.7%)	218193 (9.6%)
West Midlands	985769 (10.7%)	32583 (10.2%)	335342 (9.6%)	339441 (11.1%)	278403 (12.2%)
Yorkshire & Humber	429569 (4.7%)	17122 (5.3%)	162892 (4.6%)	139636 (4.6%)	109919 (4.8%)
Smoking					
Non smoker	5345262 (58.3%)	185677 (57.9%)	2108349 (60.1%)	1763721 (57.6%)	1287515 (56.5%)
Ex-smoker	2102775 (22.9%)	32969 (10.3%)	668375 (19.0%)	779633 (25.5%)	621798 (27.3%)
Light smoker	1204760 (13.1%)	52778 (16.5%)	520548 (14.8%)	372440 (12.2%)	258994 (11.4%)
Moderate smoker	252775 (2.8%)	10695 (3.3%)	107362 (3.1%)	78195 (2.6%)	56523 (2.5%)
Heavy smoker	119573 (1.3%)	4157 (1.3%)	46131 (1.3%)	38443 (1.3%)	30842 (1.4%)
Missing/not recorded	146379 (1.6%)	34461 (10.7%)	58448 (1.7%)	30493 (1.0%)	22977 (1.0%)

Supplementary Table 2 – Demographic characteristics by vaccination status

	Unvaccinated	One vaccine dose	Two vaccine doses	Three vaccine doses
Total sample (n)	1758689	287246	4828327	2297262
COVID-19 Deaths	10121 (0.6%)	2361 (0.8%)	1848 (<1%)	59 (<1%)
COVID-19 Hospital admission	16295 (0.9%)	2377 (0.8%)	9192 (0.2%)	4944 (0.2%)
COVID-19 Positive test	133384 (7.6%)	20780 (7.2%)	332340 (6.9%)	79957 (3.5%)
BMI, mean (SD)	25.33 (5.37)	25.71 (5.80)	26.71 (5.65)	27.68 (5.38)
Type 2 Diabetes	75888 (4.3%)	15173 (5.3%)	278274 (5.8%)	324883 (14.1%)
Type 1 Diabetes	7246 (0.4%)	1497 (0.5%)	29944 (0.6%)	21139 (0.9%)
Cardiovascular disease	75056 (4.3%)	13444 (4.7%)	186605 (3.9%)	330480 (14.4%)
Hypertension	181574 (10.3%)	32376 (11.3%)	667213 (13.8%)	911121 (39.7%)
Age, mean (SD)	43.96 (18.20)	42.14 (18.07)	47.79 (15.40)	68.09 (14.67)
Sex (Men)	919670 (52.3%)	136336 (47.5%)	2264859 (46.9%)	991458 (43.2%)
Ethnicity				
White	975521 (55.5%)	167058 (58.2%)	3231236 (66.9%)	1696838 (73.9%)
Asian	167194 (9.5%)	32276 (11.2%)	375199 (7.8%)	101545 (4.4%)
Black	132539 (7.5%)	18606 (6.5%)	137527 (2.8%)	32009 (1.4%)
Chinese	38537 (2.2%)	1695 (0.6%)	34860 (0.7%)	7295 (0.3%)
Others/not recorded	444898 (25.3%)	67611 (23.5%)	1049505 (21.7%)	459575 (20.0%)
Quintile of Townsend				
1	209124 (11.9%)	42364 (14.7%)	1220065 (25.3%)	795683 (34.6%)
2	249925 (14.2%)	49125 (17.1%)	1104953 (22.9%)	604002 (26.3%)
3	334505 (19.0%)	59086 (20.6%)	955548 (19.8%)	421198 (18.3%)
4	422064 (24.0%)	66237 (23.1%)	814316 (16.9%)	283390 (12.3%)
5	535931 (30.5%)	68758 (23.9%)	707464 (14.7%)	186042 (8.1%)
Missing/not recorded	7140 (0.4%)	1676 (0.6%)	25981 (0.5%)	6947 (0.3%)
Region				
East Midlands	78947 (4.5%)	10422 (3.6%)	212799 (4.4%)	97108 (4.2%)
East of England	79396 (4.5%)	13402 (4.7%)	279383 (5.8%)	143397 (6.2%)
London	659336 (37.5%)	79571 (27.7%)	1028853 (21.3%)	374418 (16.3%)
North East	52943 (3.0%)	9457 (3.3%)	154706 (3.2%)	77564 (3.4%)
North West	241166 (13.7%)	52651 (18.3%)	787263 (16.3%)	428724 (18.7%)
South Central	148945 (8.5%)	28277 (9.8%)	627140 (13.0%)	289618 (12.6%)
South East	126599 (7.2%)	24807 (8.6%)	500608 (10.4%)	245263 (10.7%)
South West	123909 (7.0%)	24299 (8.5%)	499147 (10.3%)	256068 (11.1%)
West Midlands	173454 (9.9%)	32847 (11.4%)	513227 (10.6%)	266241 (11.6%)
Yorkshire & Humber	73994 (4.2%)	11513 (4.0%)	225201 (4.7%)	118861 (5.2%)
Smoking				
Non smoker	944917 (53.7%)	146028 (50.8%)	2910178 (60.3%)	1344139 (58.5%)
Ex-smoker	292736 (16.6%)	45613 (15.9%)	1031504 (21.4%)	732922 (31.9%)
Light smoker	354217 (20.1%)	60439 (21.0%)	628371 (13.0%)	161733 (7.0%)
Moderate smoker	71184 (4.0%)	12960 (4.5%)	134263 (2.8%)	34368 (1.5%)
Heavy smoker	32101 (1.8%)	6043 (2.1%)	63729 (1.3%)	17700 (0.8%)
Missing/not recorded	63534 (3.6%)	16163 (5.6%)	60282 (1.2%)	6400 (0.3%)

Supplementary Table 3. Percentage of population with one, two or three vaccine doses by BMI and age group

	18-39 y		40-59 y		60-79 y		80+ y	
**Vaccine uptake**	n	%	n	%	n	%	n	%
Underweight								
No vaccine	82,731	37	13,008	26	3,987	14	4,762	27
1st dose	16,709	7	2,057	4	826	3	711	4
2nd dose	118,415	53	29,620	60	11,786	40	4,023	23
3rd dose	6,348	3	4,954	10	12,522	43	8,278	47
Healthy weight								
No vaccine	485,563	35	227,562	20	61,258	9	43,358	17
1st dose	75,419	5	30,954	3	8,741	1	6,234	2
2nd dose	773,393	56	756,679	66	275,734	39	45,944	18
3rd dose	57,965	4	131,861	12	362,439	51	166,109	63
Overweight								
No vaccine	211,811	31	186,354	16	72,538	8	42,867	14
1st dose	37,298	6	28,807	3	10,437	1	5,895	2
2nd dose	393,700	58	783,757	68	363,279	38	49,157	17
3rd dose	32,021	5	145,426	13	499,596	53	199,946	67
Obesity								
No vaccine	117,843	26	117,444	13	60,682	8	26,921	16
1st dose	26,261	6	23,159	3	10,087	1	3,615	2
2nd dose	278,547	61	601,690	67	308,963	41	33,640	20
3rd dose	31,749	7	148,687	17	382,099	50	107,262	62

Supplementary Table 4. Cox proportional hazards of vaccination across BMI groups

	Underweight		Healthy weight		Overweight		Obesity	
	HR	95%CI	HR	95%CI	HR	95%CI	HR	95%CI
1st dose	0.909	(0.905-0.913)	1		1.120	(1.118-1.122)	1.202	(1.199-1.204)
2nd dose	1.044	(1.040-1.049)	1		1.001	(0.999-1.003)	1.003	(1.001-1.005)
3rd dose	0.877	(0.867-0.887)	1		1.043	(1.040-1.047)	1.044	(1.040-1.047)

*Stratified by age (10 year bands), adjusted for age, sex, ethnicity, Townsend, region, smoking, hypertension, T1/2Diabetes, CVD, carehome status

Supplementary Table 5. Vaccine effectiveness against COVID-19 Hospital admission using a case-control study design

COVID-19 Hospital admission	Underweight					Healthy weight					Overweight					Obesity				
	N controls	N cases	OR (95%CI)			N controls	N cases	OR (95%CI)			N controls	N cases	OR (95%CI)			N controls	N cases	OR (95%CI)		
No vaccine	3729	454	1.00			42159	4291	1.00			43801	5118	1.00			31673	6432	1.00		
1st dose (interval after)																				
0-6	164	20	1.54	0.82	2.88	2986	247	1.35	1.13	1.60	3540	311	1.19	1.02	1.39	2316	292	1.02	0.88	1.20
7-13	150	35	3.38	2.06	5.55	2781	332	2.02	1.73	2.35	3480	500	2.08	1.83	2.37	2275	506	1.99	1.75	2.26
14-20	166	21	1.76	1.01	3.09	2415	277	1.74	1.48	2.04	2890	386	1.78	1.54	2.05	1895	435	1.91	1.67	2.19
21-27	129	17	1.23	0.65	2.34	1809	184	1.18	0.98	1.43	2144	180	0.80	0.66	0.97	1383	217	0.91	0.76	1.09
28+	623	63	0.67	0.48	0.94	6615	634	0.81	0.73	0.89	6670	696	0.69	0.62	0.76	4755	765	0.66	0.60	0.72
2nd dose (interval after)																				
0-6	83	<5	0.10	0.01	0.74	981	38	0.33	0.23	0.47	886	27	0.20	0.13	0.31	592	33	0.22	0.15	0.33
7-13	79	<5	0.26	0.07	0.95	1006	30	0.24	0.17	0.36	936	48	0.33	0.24	0.46	605	44	0.27	0.19	0.38
14+	2024	173	0.51	0.41	0.63	35386	2201	0.34	0.32	0.36	42116	3236	0.32	0.30	0.34	32903	4178	0.32	0.30	0.34
3rd dose (interval after)																				
0-6	35	<5	0.28	0.08	0.99	821	24	0.11	0.07	0.17	1192	38	0.09	0.06	0.12	802	39	0.08	0.06	0.11
7-13	28	<5	0.07	0.01	0.67	652	18	0.09	0.06	0.15	910	45	0.12	0.09	0.17	613	54	0.14	0.11	0.19
14+	72	<5	0.05	0.01	0.39	1398	39	0.07	0.05	0.11	1894	68	0.08	0.06	0.10	1237	49	0.05	0.04	0.07

*Cases and controls were matched by age, sex, calendar date, practice, region and carehome status; models were further adjusted for ethnicity, Townsend, smoking, hypertension, type 1 and type 2 diabetes and CVD

Supplementary Table 6. Vaccine effectiveness against COVID-19 death using a case-control study design

COVID-19 Death	Underweight					Healthy weight					Overweight					Obesity				
	N controls	N cases	OR (95%CI)			N controls	N cases	OR (95%CI)			N controls	N cases	OR (95%CI)			N controls	N cases	OR (95%CI)		
No vaccine	1145	318	1.00			17402	2684	1.00			22055	3266	1.00			15916	3865	1.00		
1st dose (interval after)																				
0-6	146	<5	0.01	0.00	1.04	2435	27	0.12	0.05	0.28	3145	23	0.05	0.02	0.13	2050	17	0.05	0.02	0.14
7-13	130	13	0.07	0.01	0.52	2213	97	0.33	0.17	0.63	2724	94	0.18	0.10	0.35	1757	76	0.12	0.06	0.24
14-20	93	22	0.23	0.03	1.53	1948	178	0.55	0.34	0.90	2413	132	0.19	0.10	0.36	1398	123	0.27	0.15	0.48
21-27	81	15	0.46	0.08	2.62	1337	140	0.46	0.26	0.83	1723	157	0.31	0.18	0.51	1089	115	0.37	0.23	0.59
28+	172	46	1.30	0.65	2.59	3503	375	0.70	0.55	0.88	4224	382	0.51	0.41	0.63	2562	330	0.37	0.29	0.45
2nd dose (interval after)																				
0-6	14	0				343	<5	0.11	0.03	0.35	419	0				234	6	0.08	0.03	0.23
7-13	23	0				323	6	0.21	0.08	0.55	398	<5	0.07	0.02	0.29	230	9	0.11	0.04	0.30
14+	252	46	0.60	0.36	0.98	5398	472	0.39	0.33	0.47	7452	606	0.30	0.25	0.35	5246	680	0.26	0.22	0.30
3rd dose (interval after)																				
0-6	6	0				195	<5	0.03	0.01	0.10	254	<5	0.02	0.00	0.07	165	0			
7-13	7	0				163	0				258	<5	0.01	0.00	0.05	134	6	0.04	0.02	0.10
14+	25	0				486	15	0.04	0.02	0.08	700	18	0.03	0.02	0.06	389	12	0.02	0.01	0.04

*Cases and controls were matched by age, sex, calendar date, practice, region and carehome status; models were further adjusted for ethnicity, Townsend, smoking, hypertension, type 1 and type 2 diabetes and CVD

Supplementary Table 7. Vaccine effectiveness against COVID-19 positive test using a case-control study design

COVID-19 Positive test	Underweight					Healthy weight					Overweight					Obesity				
	N controls	N cases	OR (95%CI)			N controls	N cases	OR (95%CI)			N controls	N cases	OR (95%CI)			N controls	N cases	OR (95%CI)		
No vaccine	108899	8944	1.00			765509	56824	1.00			467516	37766	1.00			322236	29850	1.00		
1st dose (interval after)																				
0-6	3852	730	2.59	2.38	2.82	23179	4646	3.20	3.09	3.31	15409	3093	3.08	2.96	3.22	11087	2544	3.03	2.89	3.17
7-13	4785	959	2.87	2.66	3.10	26806	6104	3.80	3.69	3.92	17001	3980	3.88	3.73	4.03	11906	3266	3.89	3.73	4.06
14-20	5073	566	1.54	1.40	1.69	28419	3705	2.09	2.01	2.17	16944	2513	2.29	2.18	2.39	11463	2166	2.54	2.41	2.67
21-27	5284	533	1.34	1.22	1.48	29067	3484	1.88	1.81	1.96	16540	2029	1.83	1.75	1.93	11078	1586	1.87	1.77	1.98
28+	29416	3996	1.83	1.76	1.91	197368	27142	2.11	2.08	2.14	122511	16793	1.98	1.94	2.02	86920	12107	1.77	1.73	1.81
2nd dose (interval after)																				
0-6	4003	376	1.23	1.10	1.37	31699	3029	1.42	1.37	1.48	19904	1919	1.35	1.29	1.42	12890	1267	1.21	1.14	1.29
7-13	3506	216	0.80	0.70	0.93	31111	2246	1.06	1.02	1.11	20376	1775	1.20	1.14	1.26	13323	1191	1.10	1.03	1.17
14+	74526	5976	1.07	1.03	1.11	1063985	105545	1.44	1.42	1.46	972248	106711	1.48	1.46	1.50	805350	90717	1.33	1.31	1.35
3rd dose (interval after)																				
0-6	582	45	0.90	0.66	1.24	14522	1214	1.09	1.02	1.15	16793	1607	1.14	1.08	1.20	14014	1406	1.06	1.00	1.12
7-13	443	19	0.43	0.26	0.70	11025	771	0.88	0.81	0.95	12751	1022	0.91	0.86	0.98	10326	847	0.83	0.78	0.90
14+	1114	43	0.45	0.33	0.61	25170	1142	0.54	0.50	0.57	26543	1172	0.47	0.44	0.50	21968	879	0.38	0.36	0.41

*Cases and controls were matched by age, sex, calendar date, practice, region and carehome status; models were further adjusted for ethnicity, Townsend, smoking, hypertension, type 1 and type 2 diabetes and CVD

Supplementary Figure 2. Risk of COVID-19 positive test after vaccination

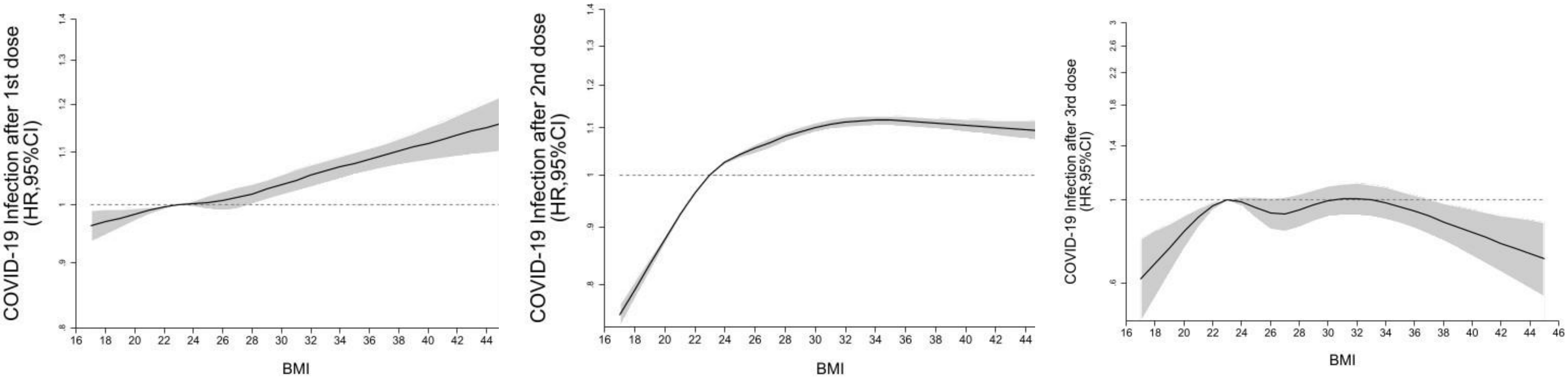

* Estimates of risk after 14 days from each vaccine dose. Adjusted for age, calendar week, sex, ethnicity, Townsend, region, smoking, hypertension, T1/2Diabetes, CVD, carehome status

Supplementary Figure 3. Sensitivity analysis of vaccine effectiveness against COVID-19 hospital admission and death among people with recent BMI measurements

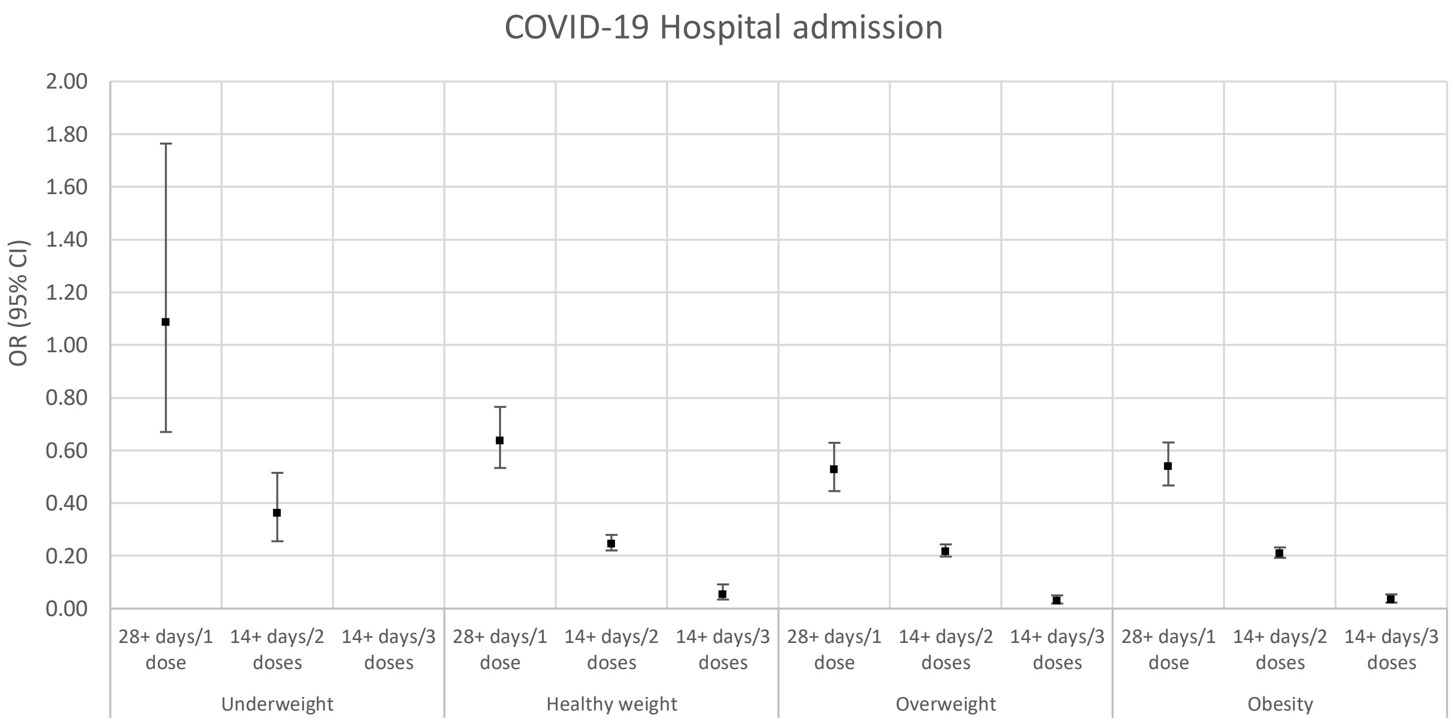

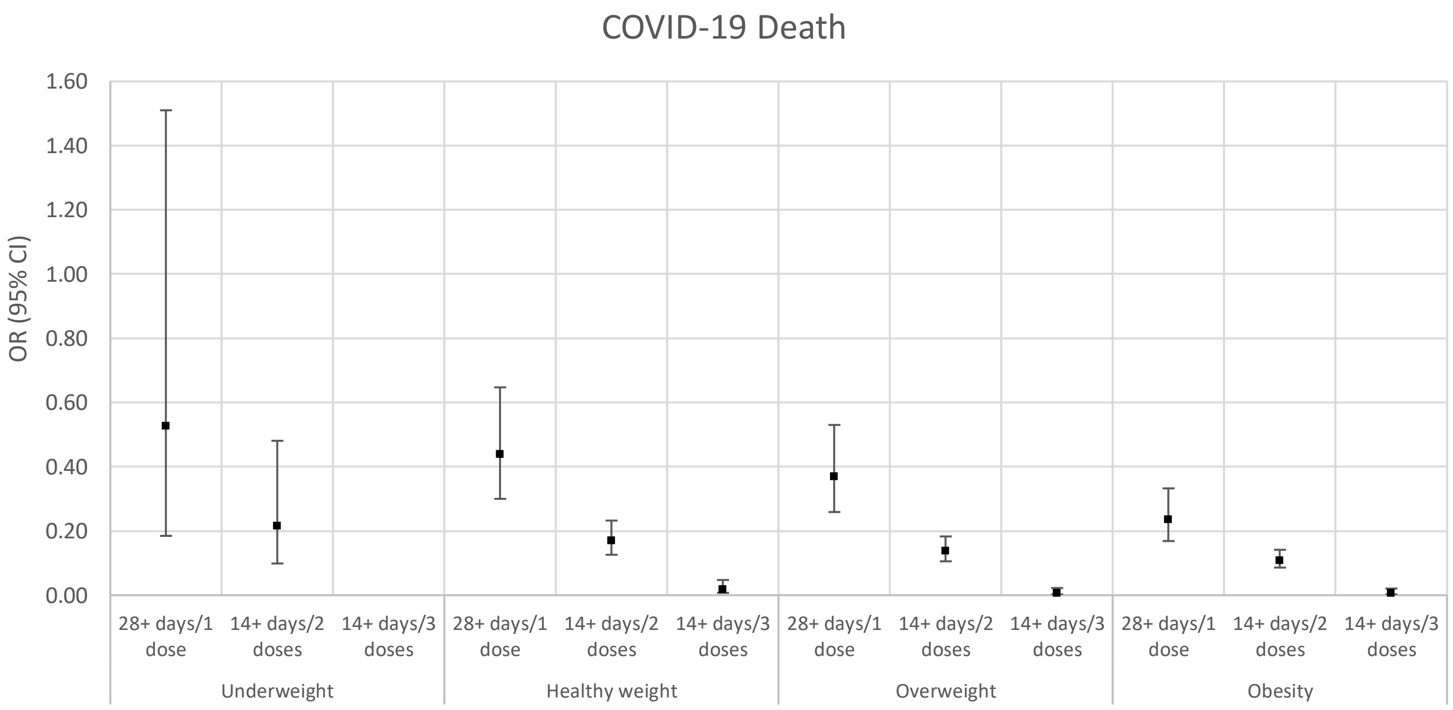

*Cases and controls were matched by age, sex, calendar date, practice, region and carehome status; models were further adjusted for ethnicity, Townsend, smoking, hypertension, type 1 and type 2 diabetes and CVD

Supplementary Figure 4. Sensitivity analysis of vaccine effectiveness against COVID-19 hospital admission and death excluding people living in care homes

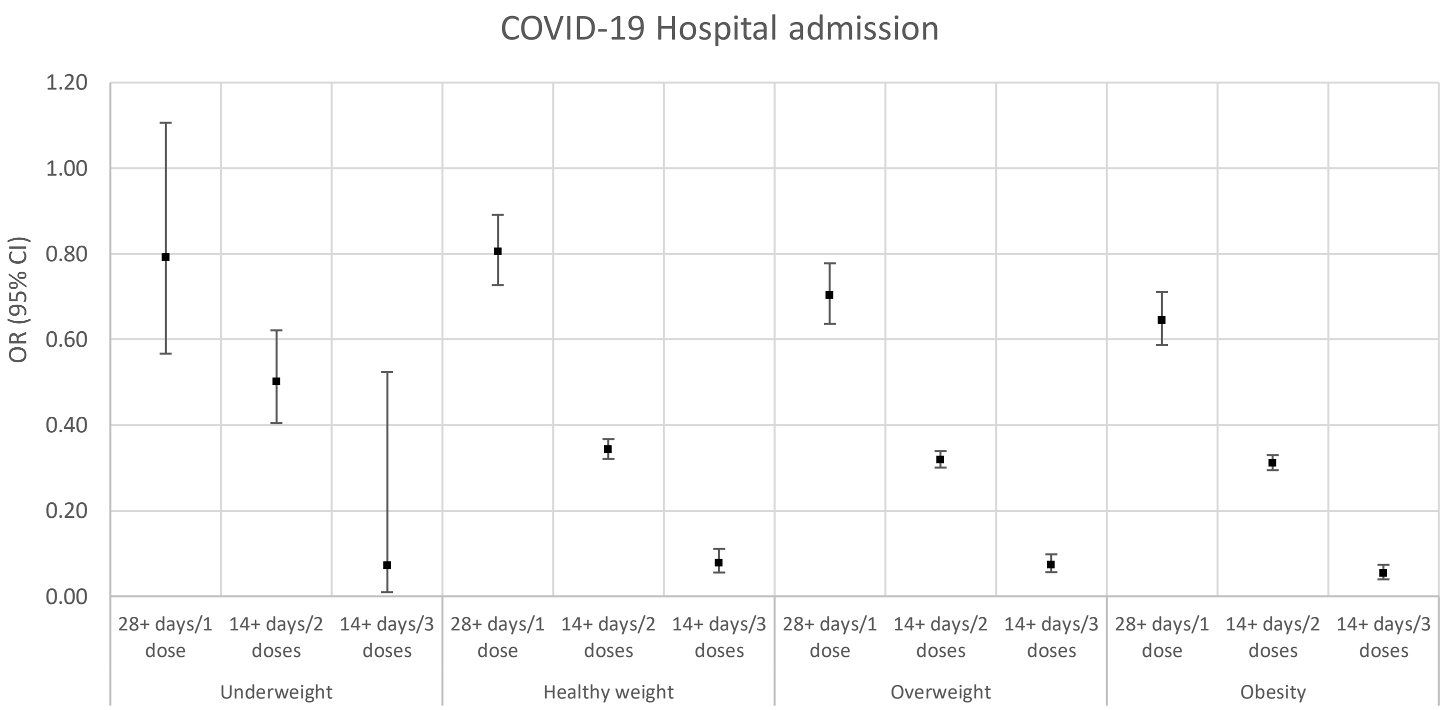

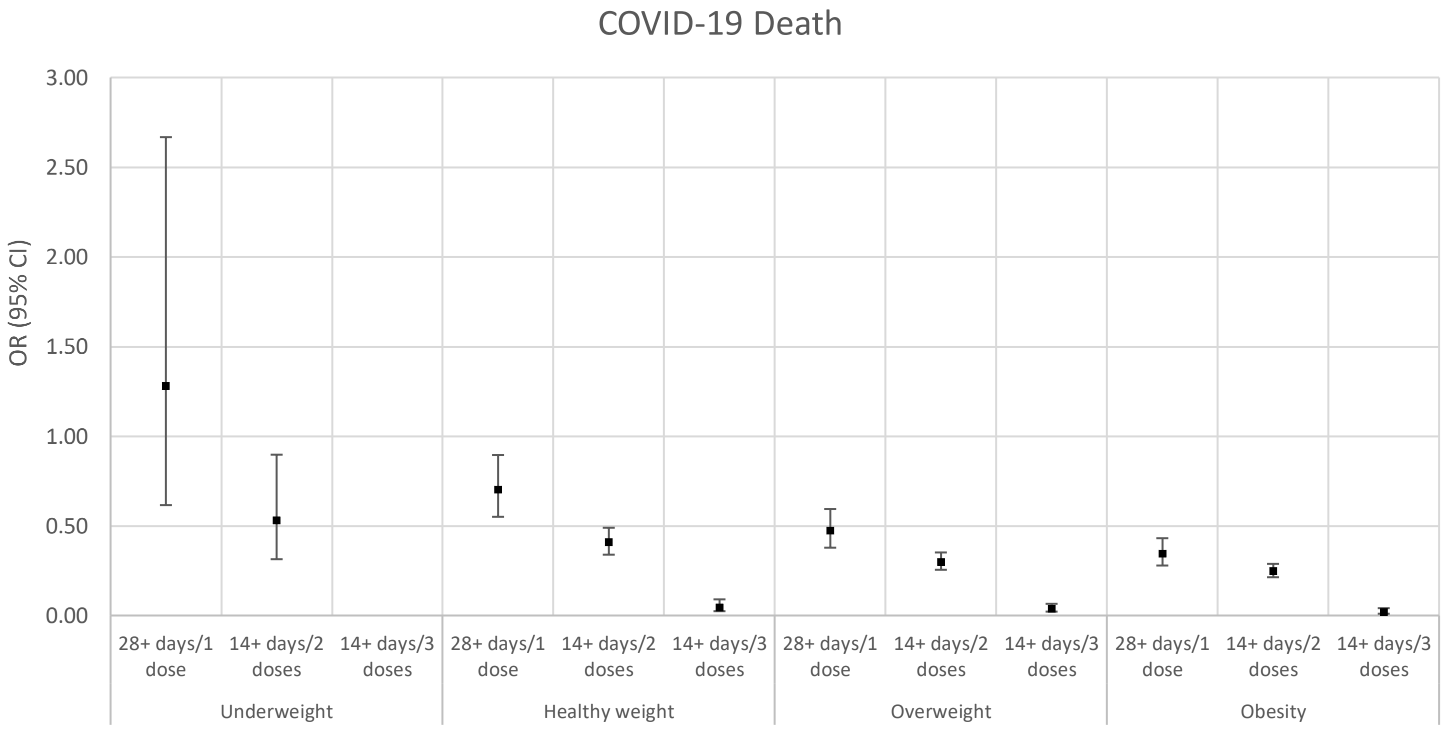

*Cases and controls were matched by age, sex, calendar date, practice, region and carehome status; models were further adjusted for ethnicity, Townsend, smoking, hypertension, type 1 and type 2 diabetes and CVD

Supplementary Figure 5. Sensitivity analysis of risk of severe COVID-19 outcomes after vaccination among people with recent BMI measurements

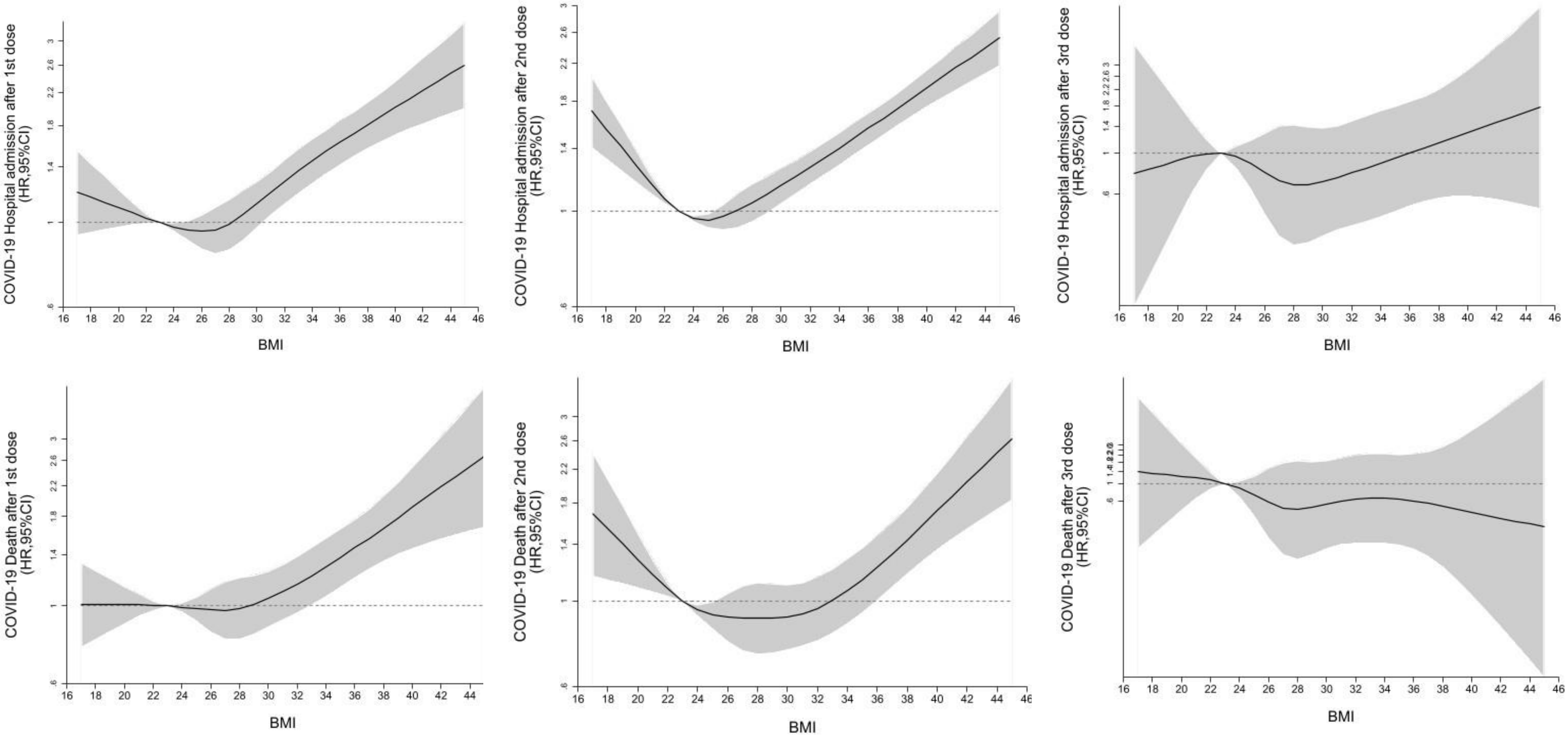

*Estimates of risk after 14 days from each vaccine dose. Adjusted for age, calendar week, sex, ethnicity, Townsend, region, smoking, hypertension, T1/2Diabetes, CVD, carehome status

Supplementary Figure 6. Sensitivity analysis of risk of severe COVID-19 outcomes after vaccination excluding people living in care homes

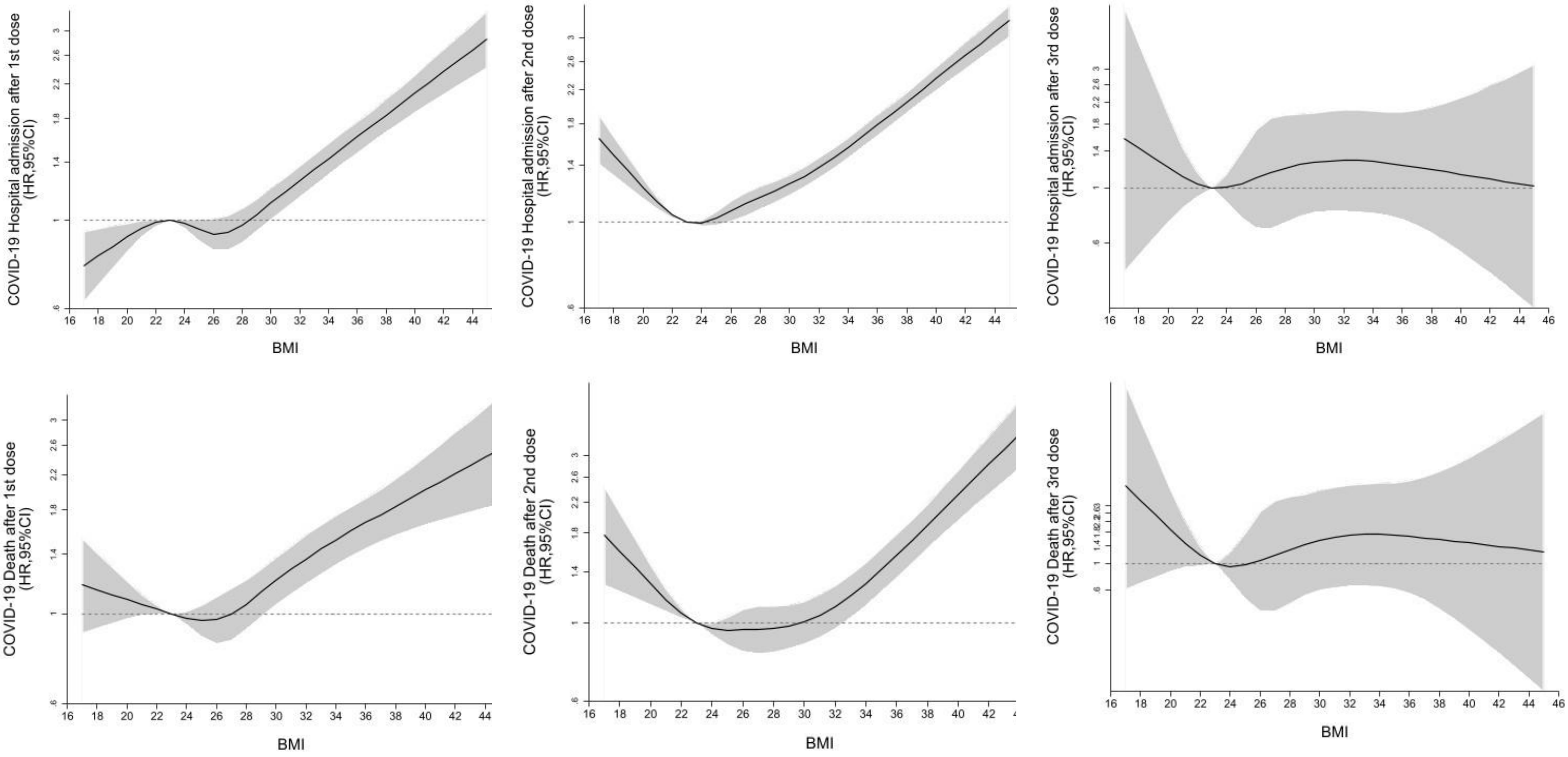

*Estimates of risk after 14 days from each vaccine dose. Adjusted for age, calendar week, sex, ethnicity, Townsend, region, smoking, hypertension, T1/2Diabetes, CVD, carehome status
