# Supplementary material for: Associations of BMI with COVID-19 vaccine uptake, vaccine effectiveness, and risk of severe COVID-19 outcomes after vaccination in England: a population-based cohort study
Source: Lancet Diabetes Endocrinol. 2022 Aug;10(8):571–80. doi: 10.1016/S2213-8587(22)00158-9 (PMC9246477; doi:10.1016/S2213-8587(22)00158-9)
Supplement: Supplementary appendix 2 [file mmc2.pdf]

THE LANCET
Diabetes & Endocrinology

Supplementary appendix 2

This appendix formed part of the original submission and has been peer reviewed. We post it as supplied by the authors.

Supplement to: Piernas C, Patone M, Astbury NM, et al. Associations of BMI with COVID-19 vaccine uptake, vaccine effectiveness, and risk of severe COVID-19 outcomes after vaccination in England: a population-based cohort study. *Lancet Diabetes Endocrinol* 2022; published online June 30. https://doi.org/10.1016/S2213-8587(22)00158-9.

Supplementary Appendix 2– Statistical Analysis Plan

Version 2.0 dated 20 December 2022

Amendment to version 1.0 dated 1 November 2021

- A new data release was received with information on vaccine doses and events up to November 17th 2021. Version 1.0 was modified to include the 3rd doses of vaccine in the analyses

Qresearch reference project OX107 - Uptake, effectiveness, and comparative safety of new COVID-19 vaccines by age, sex, region, ethnicity, comorbidities, medication, deprivation, risk level and evidence of prior COVID infection

1. Title of substudy

Uptake and effectiveness of new COVID-19 vaccines across body mass index (BMI) groups

2. Objectives

a) Examine the uptake of the COVID-19 vaccines across BMI groups
b) Examine the effectiveness of the COVID-19 vaccines across BMI groups, by evaluating the risk and severity of COVID-19 diagnosis after vaccination
c) Examine the risk of COVID-19 outcomes after vaccination in the population that has received one, two or three doses of the vaccine across the full range of BMI

3. Inclusion criteria

All patients aged 18+, with a BMI measurement in the medical record, as well as data on COVID-19 vaccination and linkage to PHE data of COVID-19 tests

4. Exclusion criteria

Patients will be excluded if:
- vaccines given before study start (8th Dec 2020);
- missing vaccine dates;
- Infection before vaccination: since only the earliest infection is available for everyone, the rationale for excluding people who were infected before vaccination is because we would not know if the outcome (infection after vaccination) occurred or not

5. Data sources and settings

Our main analyses will be based on the QResearch database linked to the following datasets to improve ascertainment of exposures, confounders and outcomes:

• Pillar 1 and 2 testing data (PHE SGSS)
• Civil registration data (NHS Digital)
• HES care data (NHS Digital)
• COVID-19 Vaccine uptake data from the National Immunisation Database (NIMS, NHS Digital)

6. Study design

a) Vaccine uptake analysis: cohort study design
b) Vaccine effectiveness analysis: nested matched case-control including both vaccinated and unvaccinated population
c) Risk of infection after vaccination: cohort study including only the vaccinated population

7. Statistical analyses

Stata 16 will be used for all analyses, and reporting of results will following STROBE guidelines for reporting of observational analyses.

Descriptive analyses will present the outcomes of interest by BMI group, as well as other key socio-demographic characteristics.

BMI (kg/m2) will be grouped into 4 categories using the WHO/NICE classification, with adjustments for Asian ethnicity:
- underweight (<18·5 kg/m2)
- normal weight (18·5–24·9 kg/m2 [or 18·5–22.9 kg/m2 for Asian ethnicity])
- overweight (25·0–29·9 kg/m2 [23–27·5 kg/m2 Asian]);
- obesity I (≥30·0 kg/m2 [≥27·5 kg/m2 Asian]);

7.1) Vaccine uptake

Outcomes:

For the vaccine uptake analyses, the main outcomes of interest are:
- At least one COVID-19 vaccination (earliest dose) administered during the study period (from 8th December 2020 (date of first vaccination in England) to the latest date for which linked data are available).
- Two and/or three doses of COVID-19 vaccine administered during the study period (from 8th December 2020 (date of first vaccination in England) to the latest date for which linked data are available).

Analysis:

- Descriptive analyses to calculate vaccine uptake (% uptake) by number of doses and BMI group in 20-year age bands (to account for the fact that not everyone was eligible for vaccine at the same time)
- Cox regression analyses to calculate adjusted hazard ratios (95% CI) for uptake of vaccination by BMI group, where healthy weight (BMI 18.5-24.9) will be the reference category, stratified by age (10-year age bands), adjusted sex, ethnicity, smoking status, deprivation, region, co-morbidity. We will check for proportional hazard and extend if appropriate to using Royston-Palmar models to account for time varying hazard ratios if the proportional hazards assumption is not valid. Patients will enter the analysis period on 8th Dec 2020 and will be censored on the date on which they leave, die or the latest date for which data are available.

7.2) Vaccine effectiveness

Outcomes:

The outcomes of interest are occurrence and severity of a COVID-19 diagnosis following one, two and three doses of COVID-19 vaccination compared to those who have not received the vaccine. However, since there will be differences in testing after vaccination (for example a high proportion of asymptomatic infections may not be tested), the hospital admission and death related outcomes will be considered more robust outcomes than infection.

- Laboratory confirmed SARS-CoV-2 infection
- COVID-19 hospitalisation
- COVID-19 related mortality

Exposure definition:

For the AZ-Oxford vaccine, the date on which patients are likely to have a significant immune response is in the second week post vaccination. However, a positive symptomatic test result in this time window is likely to reflect a virus acquired a week or so earlier. Hence patients will be considered to be not vaccinated from 8th Dec 2020 to day 14 post vaccination. For the Pfizer vaccine, patients will be considered to be unvaccinated for 8th Dec to Day 8 post vaccination, but we will use 14 days post vaccination to be consistent with the AZ-Oxford vaccine (only applicable to objective 3).

For the analysis, we will define vaccination status considering one and two doses of the vaccine and periods since vaccination as follows:

- Unvaccinated
- 0-6 days after 1st dose
- 7-13 days after 1st dose
- 14-20 days after 1st dose
- 21-28 days after 1st dose
- 28+ days after 1st dose
- 0-6 days after 2nd dose
- 7-13 days after 2nd dose
- 14+ days after 2nd dose
- 0-6 days after 3rd dose
- 7-13 days after 3rd dose
- 14+ days after 3rd dose

Analyses:

Conditional logistic regression analyses will be used to obtain ORs of the outcomes in vaccinated vs unvaccinated individuals across BMI groups.

Patients will enter the analyses on 8th Dec 2020 (date on which first vaccines became available in the UK). Each patient with COVID-19 positive test will be matched by age, sex, calendar date, practice, region and carehome status to a person without evidence of a positive test on that date, with a predetermined ratio of 1:10 cases to controls. Patients will be censored on the earliest of date of outcome of interest, death, end of the study period (last date for which data are available at the time of the analysis).

Models will be adjusted for ethnicity, deprivation, and relevant co-morbidity.

7.3) Risk of infection after vaccination

Outcomes:

The outcomes of interest are occurrence and severity of a COVID-19 diagnosis following one, two or three doses of COVID-19 vaccination compared to those who have not received the vaccine. However, since there will be differences in testing after vaccination (for example a high proportion of asymptomatic infections may not be tested), the hospital admission and death related outcomes will be considered more robust outcomes than infection.

- Laboratory confirmed SARS-CoV-2 infection
- COVID-19 hospitalisation, defined as having ICD-10 code in hospital record for either confirmed (U07.1) or suspected COVID-19 (U07.2) as primary or secondary cause, or new hospital admission associated with confirmed COVID-19 within 14 days of a positive SARS-CoV-2 test
- COVID-19 related mortality, defined using ICD-10 codes on ONS death certificates for confirmed or suspected death from COVID-19 (primary or secondary cause) within 28 days of a laboratory confirmed SARS-CoV-2 infection.

Exposure definition:

For the AZ-Oxford vaccine, the date on which patients are likely to have a significant immune response is in the second week post vaccination. However, a positive symptomatic test result in this time window is likely to reflect a virus acquired a week or so earlier. Hence patients will be considered to be not vaccinated from 8th Dec 2020 to day 14 post vaccination. For the Pfizer vaccine, patients will be considered to be unvaccinated for 8th Dec to Day 8 post vaccination, but we will use 14 days post vaccination for any doses to be consistent with the AZ-Oxford vaccine.

Analyses:

This analysis including only vaccinated people will use splines from Cox models to explore non-linear associations between BMI (continuous scale) and hazard ratios for COVID-19 outcomes 14 days after vaccination, where BMI 23 will be the reference group, and the same model specification above and covariates will be used.

8. Sensitivity analyses

1. For those people with a BMI measure taken many years prior to exposure to SARS-CoV-2, a sensitivity analysis will conducted to exclude those with BMI recorded more than 2 years before cohort entry.

2. Removal of people in care homes to reduce reverse causality
